# ﻿A new species of *Cletocamptus* Schmankevitsch, 1875 (Copepoda, Harpacticoida, Canthocamptidae) from Rayong Province, Eastern Thailand

**DOI:** 10.3897/zookeys.1151.96715

**Published:** 2023-02-28

**Authors:** Chaichat Boonyanusith, Koraon Wongkamhaeng

**Affiliations:** 1 Biology Program, Faculty of Science and Technology, Nakhon Ratchasima Rajabhat University, Nakhon Ratchasima 30000, Thailand Nakhon Ratchasima Rajabhat University Nakhon Ratchasima Thailand; 2 Department of Zoology, Faculty of Science, Kasetsart University, Bangkok 10900, Thailand Kasetsart University Bangkok Thailand

**Keywords:** Brackish water, diversity, estuary, mangrove, Southeast Asia

## Abstract

*Cletocamptusthailandensis***sp. nov.** was discovered in a water body at the base of a small mountain near the Phang Rat River Delta in Rayong Province, Eastern Thailand. The new species resembles *C.goenchim* Gómez, Ingole, Sawant & Singh, 2013 and *C.koreanus* Chang, 2013, but it can be distinguished from these two species based on the armament of the endopodal lobe of the male P5, ornamentations of the abdominal segments, the caudal ramus, the male P3Endp-3, and the relative length of the aesthetasc on the fourth segment of the female antennule. According to the combinations of certain female characteristics, including the number of setae on the P3Endp-2, the relative length of the caudal ramus, the relative length of the inner apical seta on the P3Endp-2, the shape of the P5, and the number of setae on the P3Exp-2, five groups of the *Cletocamptus* species can be defined.

## ﻿Introduction

*Cletocamptus* Schmankevitsch, 1875 has previously been considered enigmatic, as its higher taxonomic position is inconsistent, and some of its representatives exhibit a high degree of morphological variability (Gee 1999). The genus was originally placed in the family Cletodidae (e.g., Monard 1927; Lang 1936, 1948) and later in Canthocamptidae as *incertae sedis* (Por 1986). According to the shared apomorphic character of subdistal spinules on the ventral surface of the rostrum, its taxonomic position has more recently been clarified after Gómez and Yáñez-Rivera (2022) placed it, with *Cletocamptoides* (Gómez & Yáñez-Rivera, 2022) and *Amphibiperita* (Fiers & Rutledge, 1990), in the newly created subfamily Cletocamptinae of the family Canthocamptidae. Recently, 28 species has been recognized as valid, including *C.affinis* Kiefer, 1957, *C.albuquerquensis* (Herrick, 1894), *C.assimilis* Gómez & Gee, 2009, *C.axi* Mielke, 2000, *C.cecsurirensis* Gómez, Scheihing & La Barca, 2007, *C.chappuisi* Gómez, Gerber & Fuentes-Reinés, 2017, *C.confluens* (Schmeil, 1894), *C.deborahdexterae* Gómez, Fleeger, Rocha-Olivares & Foltz, 2004, *C.dominicanus* Kiefer, 1934, *C.feei* (Shen, 1956), *C.fourchensis* Gómez, Fleeger, Rocha-Olivares & Foltz, 2004, *C.goenchim* Gómez, Ingole, Sawant & Singh, 2013, *C.gomezi* Suárez-Morales, Barrera-Moreno & Ciros-Pérez, 2013, *C.gravihiatus* (Shen & Sung, 1963), *C.koreanus* Chang, 2013, *C.levis* Gómez, 2005, *C.mongolicus* Stĕrba, 1968, *C.nudus* Gómez, 2005, *C.pilosus* Gómez & Gee, 2009, *C.retrogressus* Schmankewitsch, 1875, *C.samariensis* Fuentes-Reinés, Zoppi de Roa & Torres, 2015, *C.schmidti* Mielke, 2000, *C.sinaloensis* Gómez, Fleeger, Rocha-Olivares & Foltz, 2004, *C.spinulosus* Gómez & Gee, 2009, *C.stimpsoni* Gómez, Fleeger, Rocha-Olivares & Foltz, 2004, *C.tainoi* Gómez, Gerber & Fuentes-Reinés, 2017, *C.tertius* Gómez & Gee, 2009, and *C.trichotus* Kiefer, 1929.

From a geographical viewpoint, *Cletocamptus* is considered a cosmopolitan genus that occurs across the salinity range (Boxshall and Defaye 2008), from freshwater to hypersaline environments. It generally occurs in estuaries, coastal areas, and beach lagoons on all continents. Some species, such as *C.cecsurirensis* (> 4000 m a.s.l.) and *C.gomezi* (> 2,300 m a.s.l.), have been recorded in high-altitude water bodies (Gómez et al. 2007; Suárez-Morales et al. 2013).

During the sampling of copepods in a shallow water body at the base of a small limestone mountain located near a mangrove forest approximately 7.5 km away from the Phang Rat River Delta in Rayong Province, Thailand, a species of *Cletocamptus* was discovered. The Thai *Cletocamptus* is likely identical to *C.deitersisensu*Tai and Song (1979), *C.goenchim*, recorded in India, and *C.koreanus*, recorded in Korea. However, because the Chinese *C.deitersi* has now been considered *species inquirendae* (Gómez et al. 2004; Gómez and Yáñez-Rivera 2022) and because some distinctive characters that distinguishes the Thai *Cletocamptus* from the Indian and Korean congeners was observed, the new species is justified and reported in this contribution. The description of its morphological characteristics and the results of a comparative study are presented.

## ﻿Materials and methods

**Figure 1.**
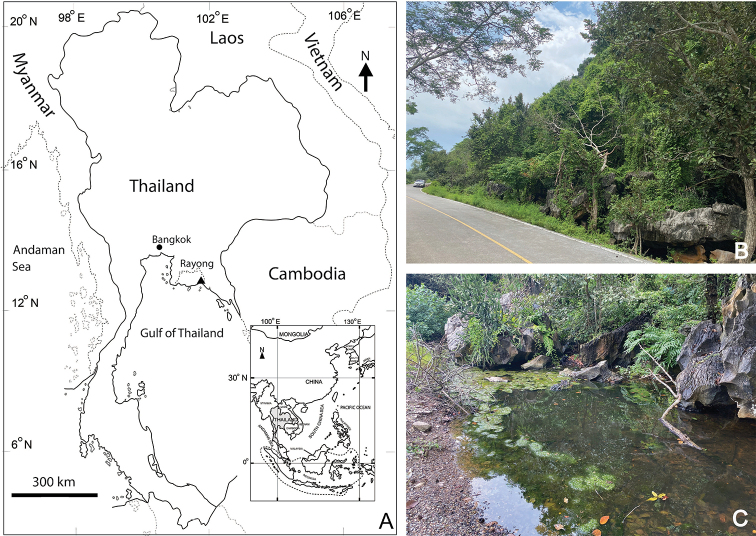
Geographical location and details of sampling site **A** map of Thailand and location of sampling site in Rayong province (indicated by a triangular) **B** details of the mountain in which, the specimens were collected **C** details of the type locality.

The sample was collected from standing water at the base of an isolated limestone mountain in the Gong Din Subdistrict of Rayong Province, eastern Thailand (Fig. 1) using a hand net with a mesh size of 60 µm, and stored in 4% formaldehyde. In the laboratory, the specimens were sorted under a stereomicroscope and stored in 70% ethanol. They were later transferred into glycerol after being placed in a mixture of glycerol and 70% ethanol (ratio ca. 1:10 v/v) for 30 min. Before morphological examination, specimens were completely dissected in a drop of 40% glycerol and mounted on a slide with coverslip.

The examination of body parts and ornamentations was performed under a Nikon ECLIPSE E200 compound light microscope at 1000× magnification. The habitus and appendages were drawn using a drawing tube attached to a compound microscope. Morphological descriptions were made based on the terminology used in Huys and Boxshall (1991). The descriptive abbreviations used in the description and figures are as follows: **ae** = aesthetasc, **I** = spine, **Endp** = endopod, **Exp** = exopod, **Endp-1 (2)** = proximal (distal) segment of the endopod of the swimming legs, **Exp-1 (2, 3)** = proximal (middle, distal) segment of the exopod of the swimming legs, **P1–P6** = first to sixth swimming legs, **Seta I–VII** = first to seventh caudal seta, **Seta I** = anterolateral accessory seta, **Seta II** = anterolateral seta, **Seta III** = postereolateral seta, **Seta IV** = outer terminal seta, **Seta V** = inner terminal seta, **Seta VI** = terminal accessory seta, and **Seta VII** = dorsal seta.

The type materials have been deposited at the Princess Maha Chakri Sirindhorn National History Museum at Prince of Songkla University, Songkhla, Thailand (**PSUNHM**).

## ﻿Taxonomy

### ﻿Order Harpacticoida G.O. Sars, 1903

**Family Canthocamptidae Brady, 1880**

**Subfamily Cletocamptinae Gómez & Yáñez-Rivera, 2022**

#### ﻿Genus *Cletocamptus* Schmankevitsch, 1875

##### ﻿ Cletocamptus thailandensis sp. nov.

Taxon classificationAnimaliaHarpacticoidaCanthocamptidae

3F794CB1-1F5A-5A4B-A056-49DFA5E4C01C

https://zoobank.org/31517B17-C7FE-484F-B96B-69F54CBA879E

**Figure 2.**
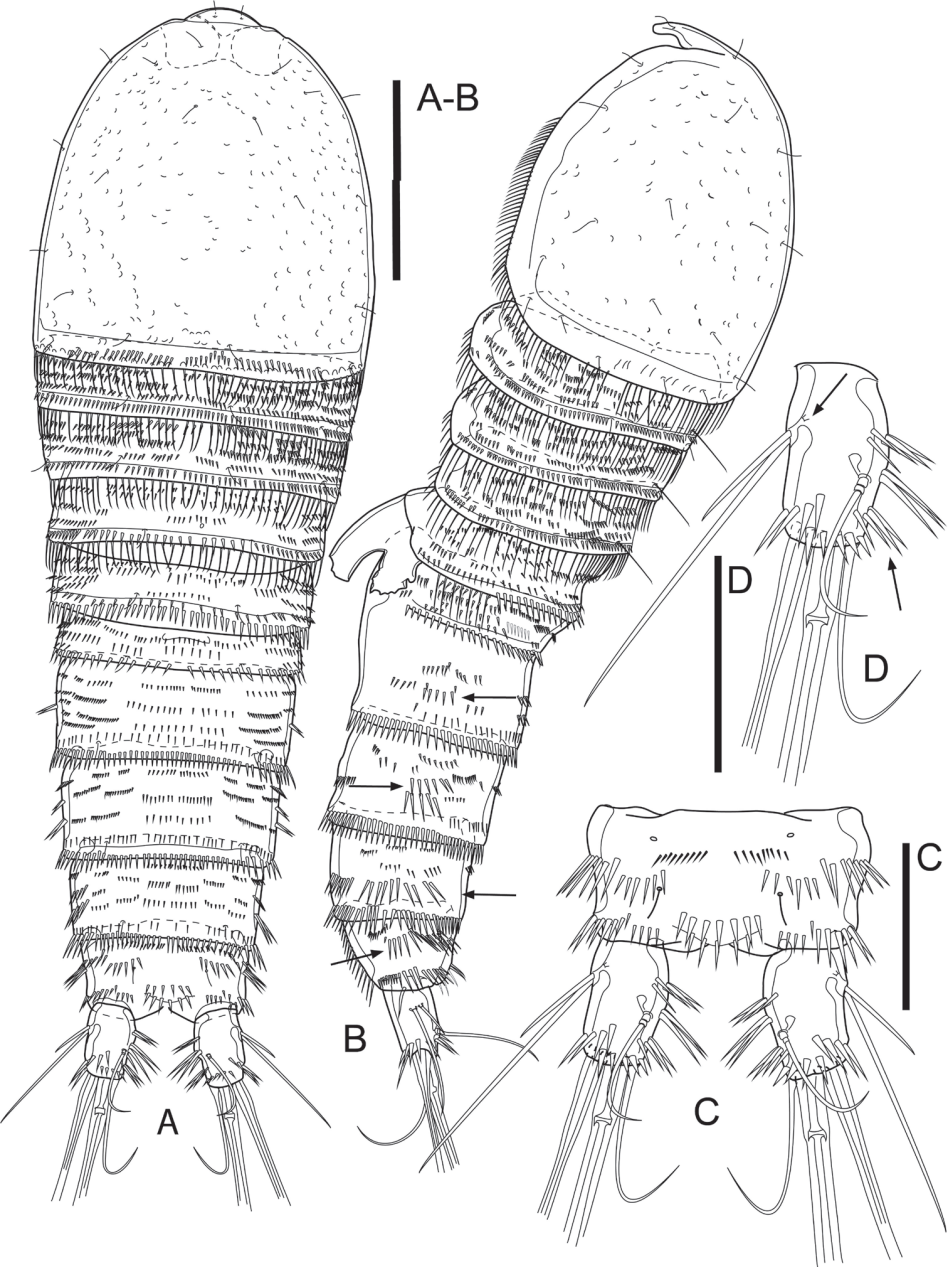
*Cletocamptusthailandensis* sp. nov. female, holotype **A** habitus, dorsal view **B** habitus, lateral view (Arrows indicate rows of moderately long spinules) **C** anal somite and caudal rami, dorsal view **D** caudal ramus, dorsal view (Arrows indicate cuticular pore and additional row of spinules). Scale bars: 50 μm.

**Figure 3.**
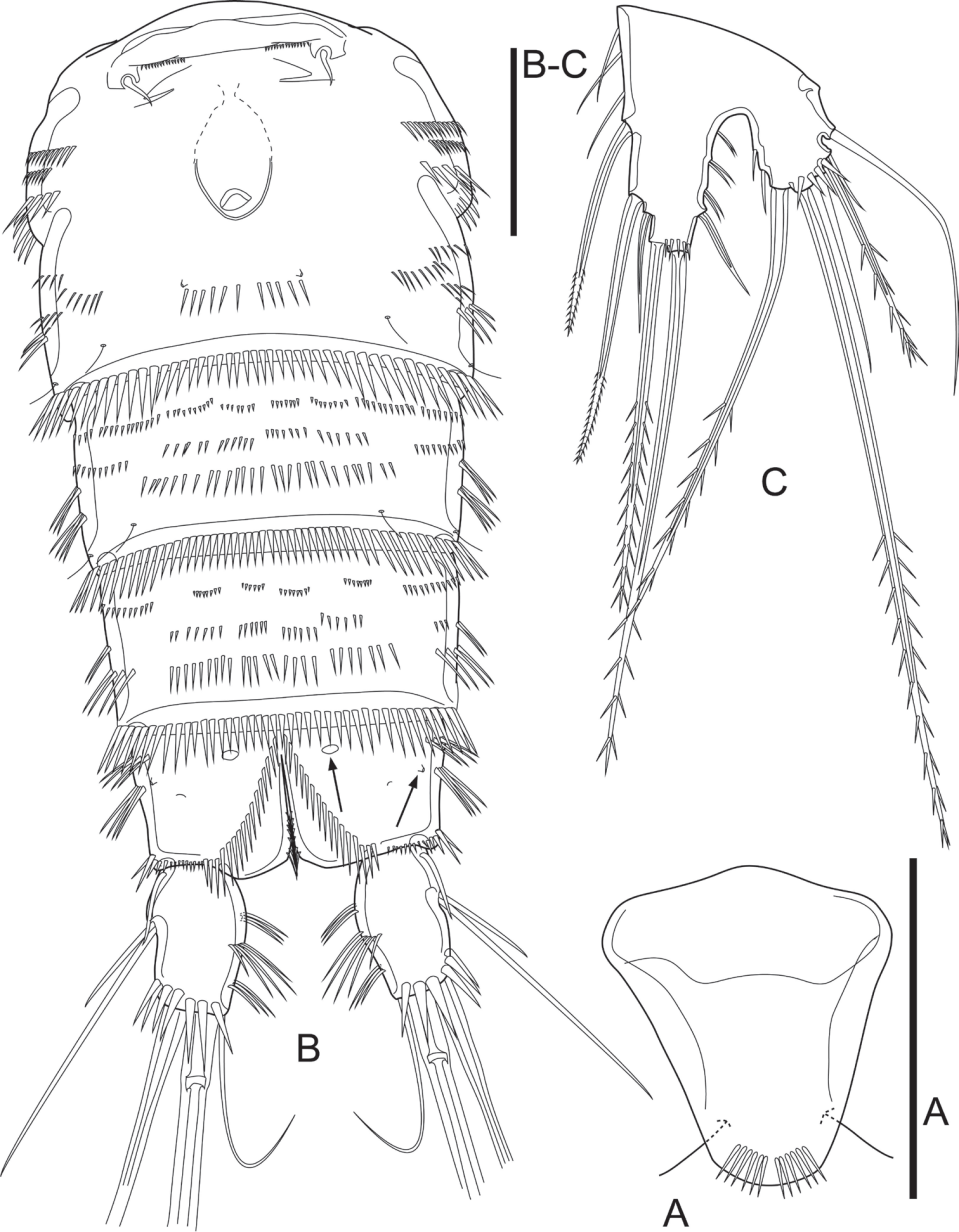
*Cletocamptusthailandensis* sp. nov. female, holotype **A** rostrum, ventral view **B** urosome, ventral view **C** P5. Scale bars: 50 μm.

**Figure 4.**
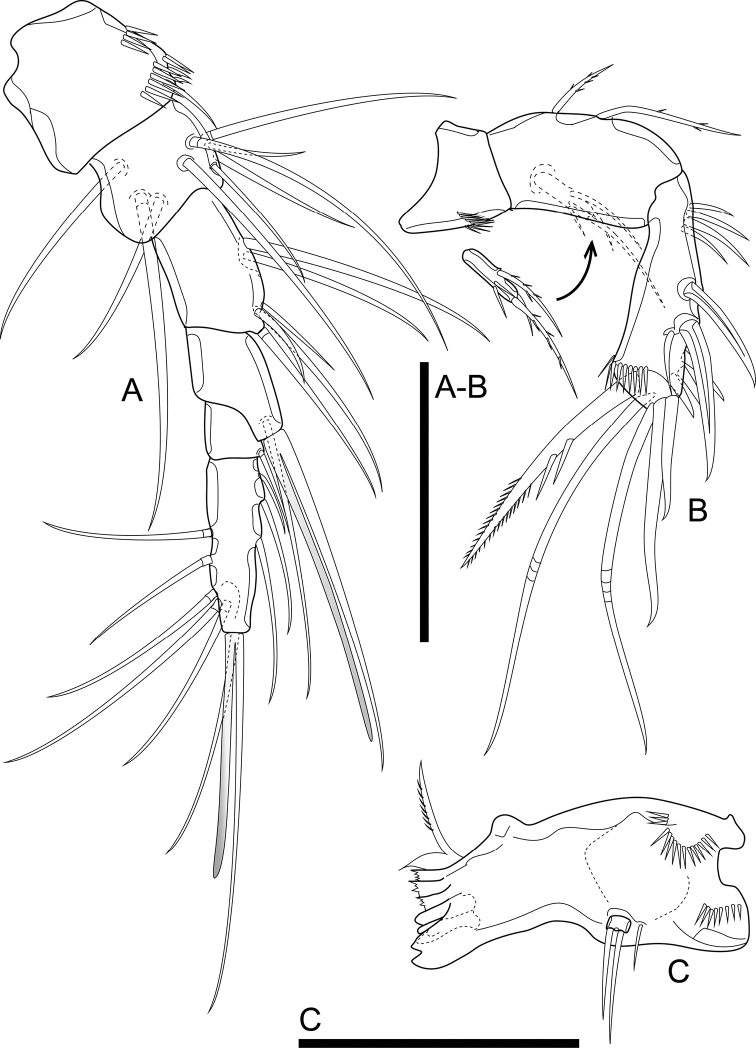
*Cletocamptusthailandensis* sp. nov. female, holotype **A** antennule **B** antenna **C** mandible. Scale bars: 50 μm.

**Figure 5.**
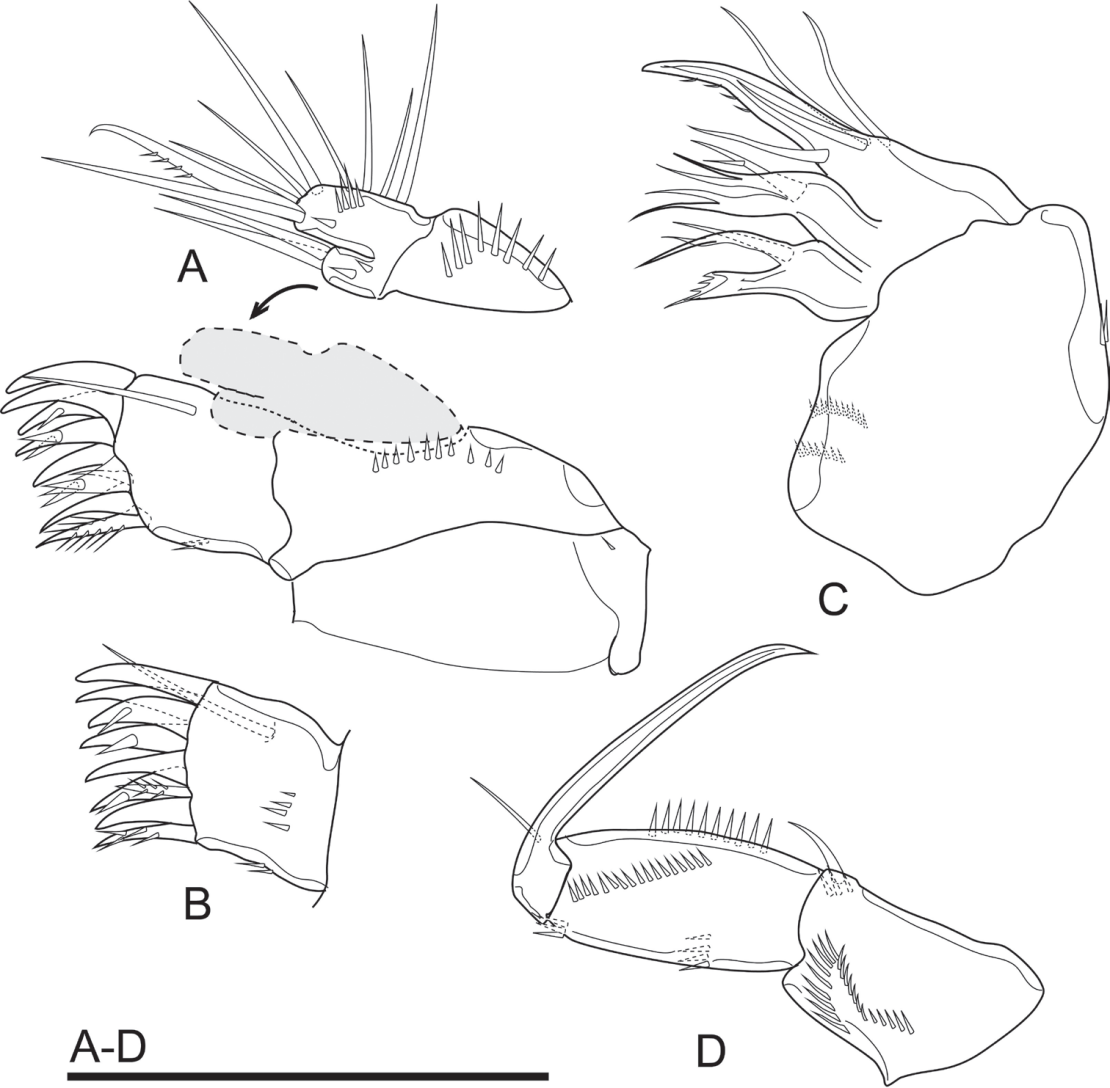
*Cletocamptusthailandensis* sp. nov. female, holotype **A** maxillule, frontal view **B** praecoxal arthrite, caudal view **C** maxilla, caudal view **D** maxilliped, caudal view. Scale bar: 50 μm.

**Figure 6.**
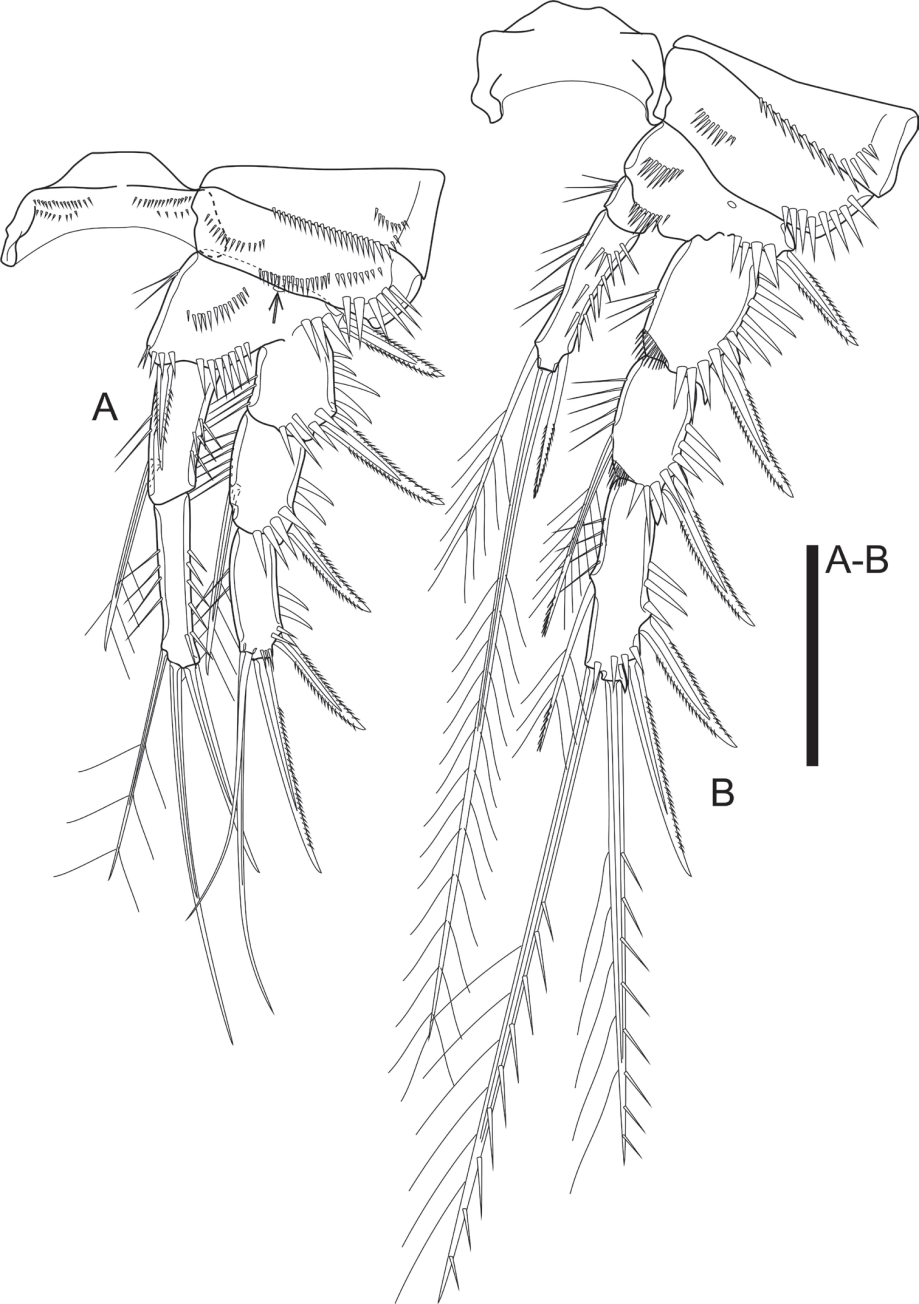
*Cletocamptusthailandensis* sp. nov. female, holotype **A** P1 **B** P2. Scale bar: 50 μm.

**Figure 7.**
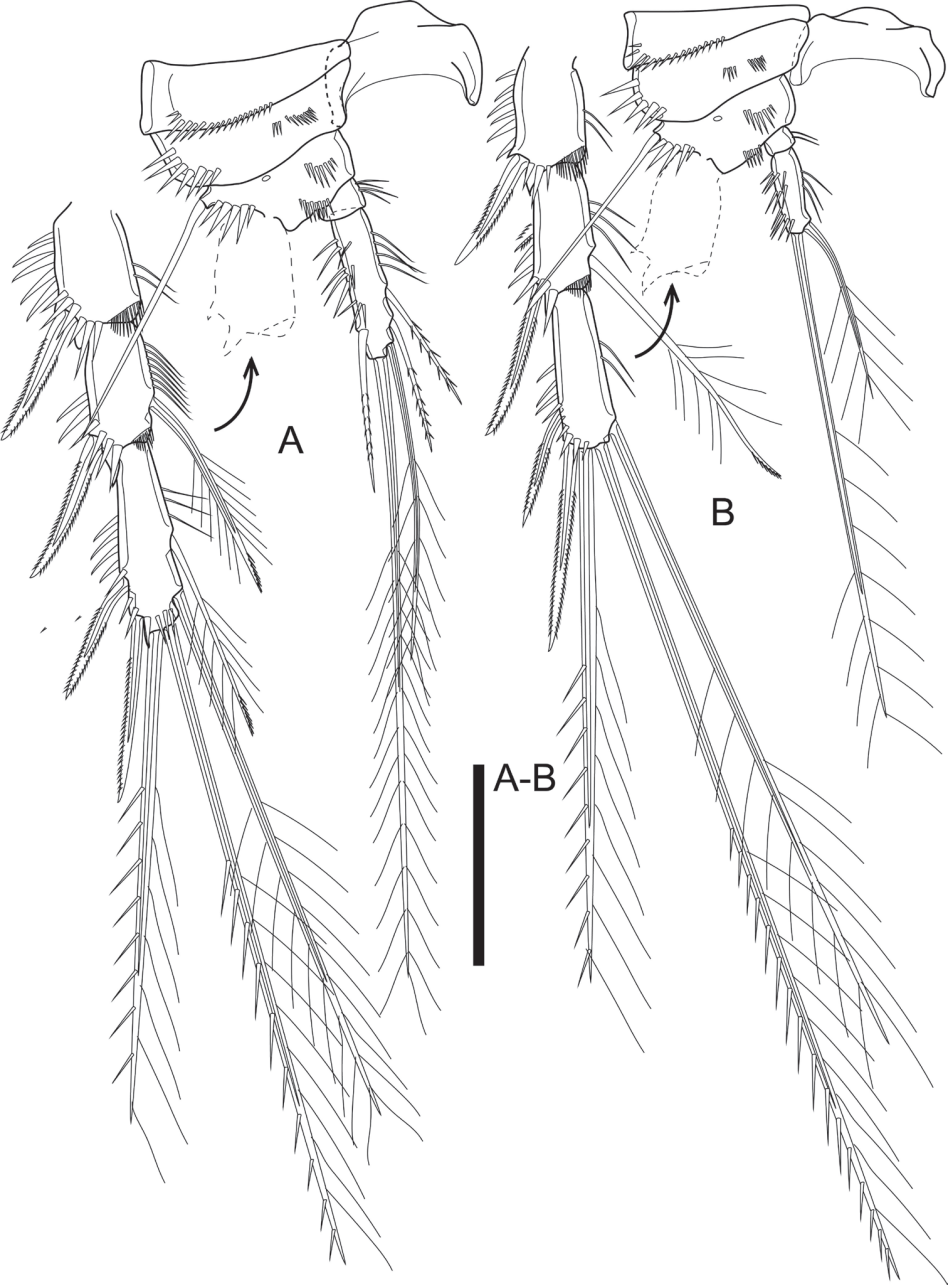
*Cletocamptusthailandensis* sp. nov. female, holotype **A** P3 **B** P4. Scale bar: 50 μm.

Figs 2 , 3 , 4 , 5 , 6 , 7 (female), 8–11 (male)

###### Material examined.

***Holotype***: Thailand • 1 ♀ (adult), 670 μm long; Rayong Province; 12°45'52.96"N, 101°47'55.16"E, 24 m a.s.l.; 19 Jul. 2022; C. Boonyanusith leg.; hand net; completely dissected and mounted on a slide in glycerol and sealed with nail polish; PSUZC-PK2007-01. ***Allotype***: Thailand • 1 ♂ (adult), 483 μm long; 22 May 2022; other collection data as for holotype; PSUZC-PK2007-02. ***Paratypes***: Thailand • 1 ♀ (adult) and 1 ♂ (adult); same data as for holotype; PSUZC-PK2007-03 and PSUZC-PK2007-04, respectively.

###### Additional material.

Thailand • 1 ♂ (adult), 1 ♀ (adult); same data as for holotype; preserved in 70% ethanol; retained in collection of the first author (CB).

###### Diagnosis.

**Female**: Body fusiform. All abdominal somites with row of moderately long spinules on lateral surface. Anal operculum with two rows of spinules. Caudal ramus ca. 1.7× as long as wide, with three rows of spinules on inner margin. Antennary exopod with three setae. Mandibular palp with two setae on free segment and one seta arising nearby. Praecoxal arthrite of maxillule with moderately strong, lateral seta. P1Endp-1 reaching distal fourth of Exp-2, with inner seta. Armature complement of Exp-3 and Endp-2, from P1–P4: 4.5.6.5 and 3.3.5.2, respectively. Inner apical seta on P3Endp-3 reaching mid length of outer one. P5 with large notch between baseoendopodal lobe and exopodal one, with six marginal setae on the former and five setae on the latter. P6 reduced to small protuberance with one apical seta on peduncle. **Male**: Left and right legs of P5 fused at base, with three marginal setae on baseoendopodal lobe and four setae on exopodal one. P6 reduced to simple unarmed plate.

###### Description of adult female.

Total body length, excluding caudal setae, ranging from 630 µm to 680 µm (mean = 655; *n* = 4). Habitus tapering posteriorly, with maximum width at posterior fourth of cephalothorax (Fig. 2A, B). Rostrum (Fig. 3A) well developed, distinct, with broad base and rounded tip; dorsal surface with pair of sensilla; ventral surface with arch row of long spinules subdistally. Prosome ca. 1.1× as long as urosome (including caudal rami), comprising cephalothorax and three free pedigerous somites (P2–P4-bearing somites). Cephalothorax ca. 1.1× as long as wide and ca. 0.5× as long as length of prosome, furnished with numerous pits, with numerous sensilla and with long spinules along margin dorsally and laterally (Fig. 2A, B). Dorsal and lateral surfaces of free pedigerous somites with numerous transverse rows of spinules, with stronger spinules and subdistal sensilla near posterior margin; posterior margin with long spinules.

Urosome (Figs 2A, B, 3B) comprising fifth pedigerous somite (P5-bearing somite), genital double-somite, and three free abdominal somites; lateral surface of all abdominal somites with row of moderately long spinules (Fig. 2B). Fifth pedigerous somite (first urosomite) with numerous transverse rows of spinules, with spinules and subdistal sensilla near posterior margin; posterior margin with larger spinules dorsally and long spinules laterally. Second and third urosomites fused ventrally forming genital double-somite, ca. 0.6× as wide as long, with subchitinous rib representing former division of genital somite and first abdominal one and dividing genital double-somite into anterior and posterior portions; dorsal and lateral surfaces of both portions with numerous transverse rows of spinules, with spinules along margin of subchitinous rib and with hair-like spinules near posterior margin of posterior portion; ventral surface with row of spinules medially, with ventral pore beside medial row of spinules (Fig. 3B); posterior margin of double-somite with moderately long spinules. Genital field (Fig. 3B) with ovipore medially at the middle of genital double-somite. Second and third abdominal somites (fourth and fifth urosomites) with numerous transverse rows of spinules, with hair-like spinules dorsally and sensilla near posterior margin and with moderately longer spinules along posterior margin.

Anal somite (Figs 2C, 3B) with pair of dorsal sensilla in front of anal operculum, with numerous paired rows of moderately long spinules on dorsal, lateral, and ventral surfaces as shown, accompanied with two pairs of cuticular pores ventrally. Anal operculum slightly convex, with two rows of spinules.

Caudal rami (Figs 2C, D, 3B) slightly divergent, each ca. 1.7× as long as wide, with seven setae; ornamentation comprised of three transverse rows of spinules on inner margin, row of spinules dorsally near distal margin, oblique row of strong spinules near insertion of seta IV, row of long spinules ventrally near insertion of seta IV and seta V, and cuticular pore near base of seta II. Seta I inserted ventro-laterally near insertion of rami; seta II and seta III inserted closely to each another at anterior third of ramus; seta IV and V, ca. 0.22 and ca. 0.58 of body length, respectively, with fracture plane in seta V; seta VI slender, inserted on inner distal corner; seta VII biarticulate, inserted at mid length of ramus medially.

Antennule (Fig. 4A) six-segmented. First segment with two rows of spinules medially. Fourth segment with aesthetasc on peduncle; aesthetasc slightly elongated, > 45% of length of aesthetasc surpassing the tip of antennule. Ultimate segment ca. 4.5× as long as wide, with aesthetasc. Aesthetascs fused to seta at base. Armature formula: 1-[1], 2-[9], 3-[6], 4-[1 + (1 + ae)], 5-[1], 6-[9 + (1 + ae)].

Antenna (Fig. 4B) biramous, comprising coxa, allobasis, one-segmented Exp, and one-segmented Endp. Coxa short, with arch row of spinules on outer margin. Allobasis with two abexopodal setae. Exp cylindrical, with few spinules subdistally and armed with one lateral seta and two apical setae (one of the apical setae short and slim). Endp club-shaped, with one short seta and two spines medially on inner margin, and five apical setae of which two innermost ones spiniform, two median ones geniculate, outermost one spiniform with two strong spinules half-length of element and bipinnate along distal half; spinule ornamentation comprised of proximal and distal rows of strong spinules, and row of spinules distally.

Mandible (Fig. 4C) comprising sclerotized coxal gnathobase and mandibular palp. Coxal gnathobase with two arch rows of spinules as shown; cutting edge with five bicuspidate and multicuspidate teeth, with one pyriform element and pinnate, ventral seta. Mandibular palp one-segmented, with two apical setae, accompanied with one short seta inserted nearby.

Maxillule (Fig. 5A, B) comprising robust praecoxa, coxa, and basal complex, the latter composed of basis with Endp and Exp completely incorporated to the latter. Praecoxal arthrite with row of spinules on caudal surface and on lateral margin, armed with surface seta on frontal surface and nine distal elements: seven of which strong, curved spines, subdistal seta slim and pinnate, lateral seta moderately strong and pinnate. Coxa with cylindrical endite bearing two smooth apical setae, and with few spinules. Basis seemingly with three apical setae of which middle robust and pinnate. Endp and Exp completely incorporated to basis, seemingly represented by three setae each.

Maxilla (Fig. 5C) composed of syncoxa and allobasis, and Endp fully incorporated to the latter. Syncoxa with two endites, each endite armed with three elements apically, one of which slender. Allobasis drawn out into claw, with one seta basally on caudal surface. Endp completely incorporated to allobasis, represented by three smooth setae.

Maxilliped (Fig. 5D) subchelate, three-segmented, comprising syncoxa, basis, and Endp. Syncoxa with curved row of spinules on caudal surface, with one pinnate seta on inner distal corner. Basis with longitudinal row of spinules on frontal and caudal surfaces, and two transverse rows of spinules on outer margin. Endp drawn out into strong claw and armed with one minute seta near base.

P1–P4 comprised of intercoxal sclerite, praecoxa, coxa, basis, and two rami.

P1 (Fig. 6A). Intercoxal sclerite as shown, with two rows of minute spinules on each side. Praecoxa triangular, with spinules along distal margin and on outer distal corner. Coxa rectangular, with one row of spinules medially, with two oblique rows of spinules and with strong spinules on outer distal corner. Basis with integumental pore on frontal surface, with oblique row of spinules and three rows of stronger spinules of which innermost at base of inner spine, median between insertion of rami, outermost at base of Exp; armament comprising outer and inner spines, inner spine reaching distal third of Endp-1. Exp three-segmented, all segments with spinule row on outer margin and outer distal corner; Exp-1 with outer spine; Exp-2 with outer spine and inner seta; Exp-3 with four elements: innermost and inner apical ones slender. Endp two-segmented, reaching tip of Exp; Endp-1 reaching distal fourth of Exp-2, with inner seta; Endp-2 slightly longer than Endp-1, with three elements of which outer apical spiniform.

P2 (Fig. 6B). Intercoxal sclerite as shown, bare. Praecoxa with spinules along distal margin. Coxa with row of spinules medially and strong spinules along outer margin. Basis as that of P1 but lacking inner spine and inner distal corner; spinule row between rami with smaller spinules relative to those of P1; with medial pore proximally. Exp three-segmented, with spinule ornamentation as that of P1, additionally with row of spinule on inner distal corner of Exp-1 and Exp-2; Exp-1 with outer spine; Exp-2 with outer spine and inner seta; Exp-3 with five elements (two outer spines, two distal and one inner seta). Endp two-segmented, reaching middle of Exp-2; Endp-1 small, wider than long, with inner spinules; Endp-2 ca. 3.0× as long as wide, with spinule ornamentation on outer and inner margins, armed with three elements of which outer spiniform and slender.

P3 (Fig. 7A). Intercoxal sclerite, praecoxa, coxa, and basis as those of P2. Exp three-segmented; Exp-1 and Exp-2 as those of P2; Exp-3 with six elements (two outer spines, two distal and two inner seta). Endp two-segmented; Endp-1 small, wider than long, with inner spinules; Endp-2 ca. 3.5× as long as wide, armed with five elements of which outer spiniform and slender, inner apical one reaching middle of outer apical one, two inner ones subequal in length.

P4 (Fig. 7B). Intercoxal sclerite, praecoxa, coxa, and basis as those of P2. Exp three-segmented; Exp-1 and Exp-2 as those of P2; Exp-3 with five elements (two outer spines, two distal and one inner seta). Endp two-segmented; Endp-1 small, wider than long, with inner spinules; Endp-2 ca. 2.5× as long as wide, armed with two apical setae of which outer ca. 2.5× as long as inner one.

Armature formula of P1–P4 as in Table 1.

**Table 1.** Armature formula of P1–P4 of *Cletocamptusthailandensis* sp. nov. Arabic numerals indicate number of setae; Roman numerals indicate number of spines.

Legs	Basis	Exopod			Endopod		
		1	2	3	1	2	3
P1	I-I	I-0	I-1	I-I1-1	0-1	0-I1-1	
P2	I-0	I-0	I-1	II-2-1	0-0	I-1-1	
P3	1-0	I-0	I-1	II-2-2	0-0	I-2-2	
P3 (male)	1-0	I-0	I-1	II-2-2	0-0	0-0	0-2-0
P4	1-0	I-0	I-1	II-2-1	0-0	0-2-0	

P5 (Fig. 3C). Baseoendopod and Exp completely fused basally, with large notch between them; left and right legs completely separated. Baseoendopodal lobe ca. 1.5× as long as length of exopodal one, with one outer, two apical and three inner setae. Exopodal lobe with five elements accompanied with slender, outer seta of basis; relative length and characteristics of setae on both baseoendopodal and exopodal lobes as shown.

P6 (Fig. 3B) reduced to minute prominence, forming simple plate near anterior margin of genital double-somite, with row of minute spinules and one short seta on each side.

###### Description of adult male.

**Figure 8.**
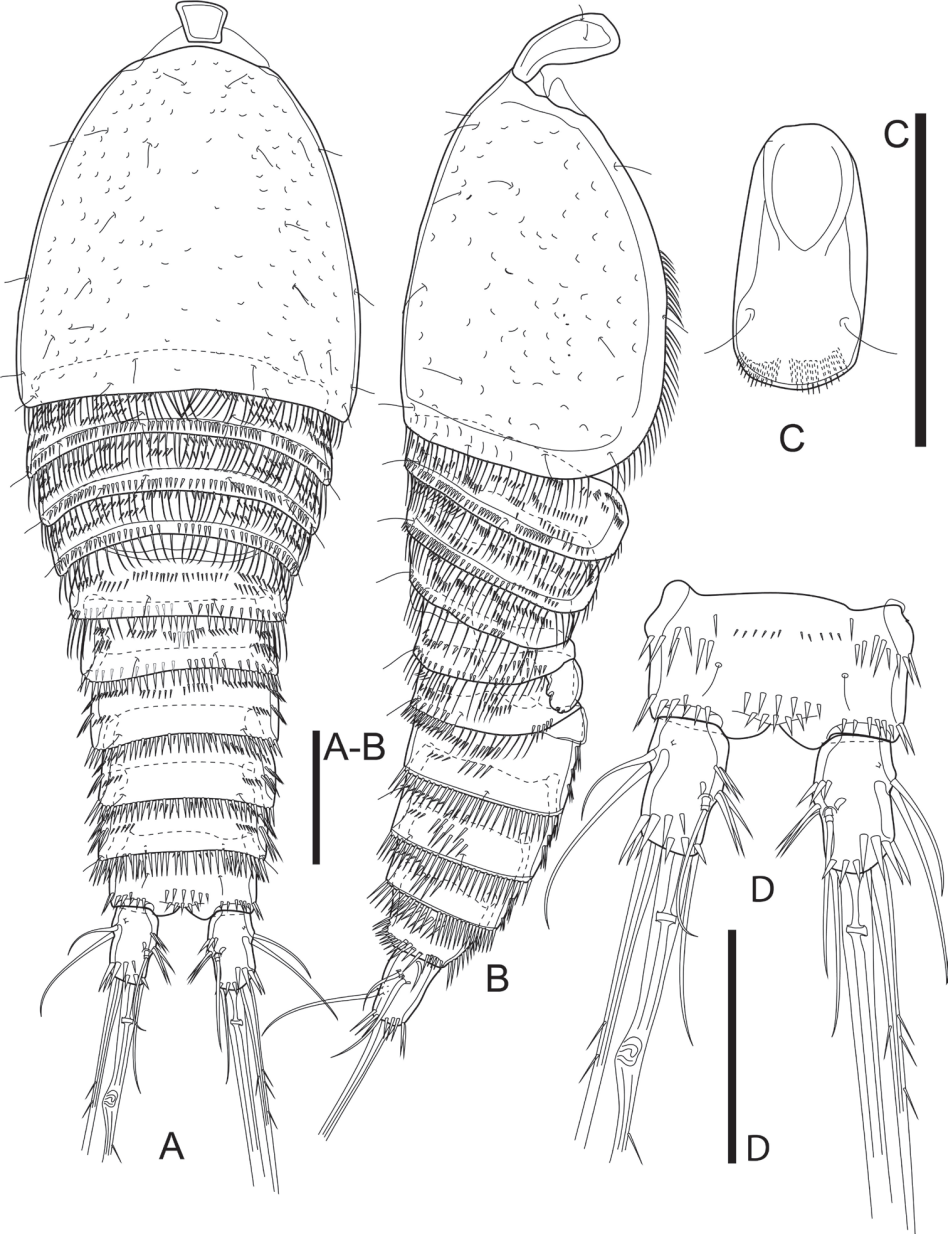
*Cletocamptusthailandensis* sp. nov. male, allotype **A** habitus, dorsal view **B** habitus, lateral view **C** rostrum, frontal view **D** anal somite and caudal rami, dorsal view. Scale bars: 50 μm.

Body smaller than in female. Total body length, excluding caudal setae, ranging from 483 µm to 505 µm (mean = 493; *n* = 4). Prosome ca. 1.1× as long as urosome (Fig. 8A, B). Cephalothorax slightly longer than wide, ca. 0.6× as long as length of prosome. Rostrum (Fig. 8C) well developed, distinct, narrower than that of female, with rounded tip; dorsal surface with pair of sensilla, ventral surface with arch row of long spinules subdistally. Ornamentation of cephalothorax and free prosomite as those of female.

**Figure 9.**
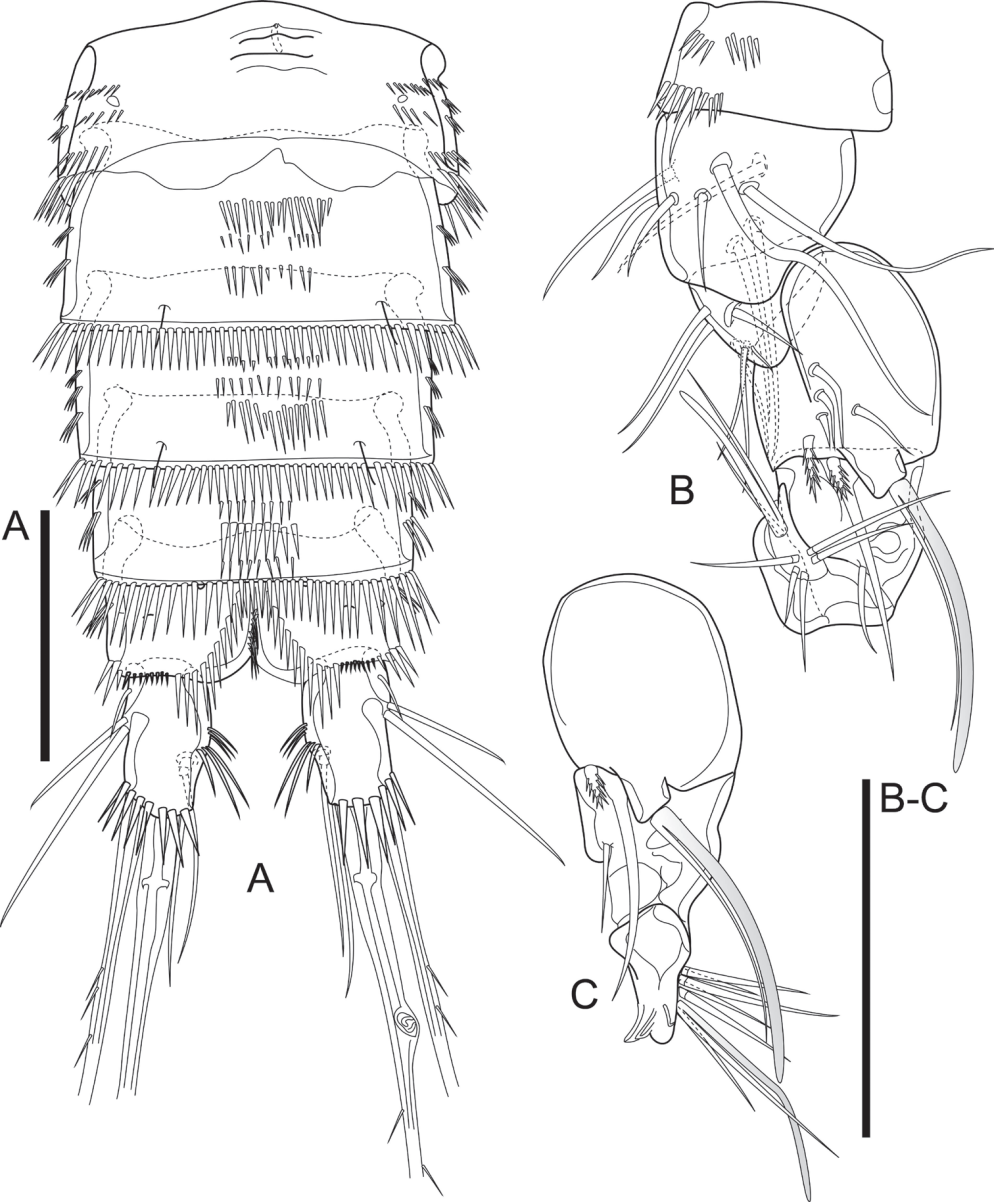
*Cletocamptusthailandensis* sp. nov. male, allotype (**A, B**); paratype (**C**) **A** urosome, ventral view **B** antennule **C** segments 4–6 of antennule. Scale bars: 50 μm.

Urosome (Figs 8A, B, 9A) six-segmented, comprising fifth pedigerous somite, genital somite and four free abdominal somites; lateral surface of all abdominal somites with row of moderately long spinules. Dorsal and lateral surfaces of fifth pedigerous somite with numerous transverse rows of spinules, with spinules and subdistal sensilla near posterior margin; posterior margin with long spinules. Genital somite with numerous transverse rows of spinules, with spinules and subdistal sensilla near posterior margin; posterior margin with larger spinules dorsally and long spinules laterally. Genital opening positioned mid-ventrally near anterior margin of genital somite (Fig. 9A). First to third abdominal somites as those of female, but lacking hair-like spinules near posterior margin; spinule ornamentation lesser developed in comparison to those of female, each somite with three medial rows of spinules ventrally. Anal somite (Figs 8D, 9A) as that of female.

Caudal rami (Figs 8D, 9A) as that of female, but inner margin with only two transverse rows of spinules. Seta IV and V, ca. 0.26× and ca. 0.59× body length, respectively. Seta VI relatively shorter than that of female.

Antennule (Fig. 9B, C) subchirocerate, six-segmented. First segment with two rows of spinules medially. Fourth segment bulbous, with aesthetasc on peduncle. Ultimate segment with two claw-shaped extensions subdistally. Aesthetascs to seta at base. Armature formula: 1-[1], 2-[9], 3-[6], 4-[7 + (1 + ae)], 5-[1], 6-[6 + (1+ae)].

Antenna, mandible, maxillule, maxilla, and maxilliped as those of female.

**Figure 10.**
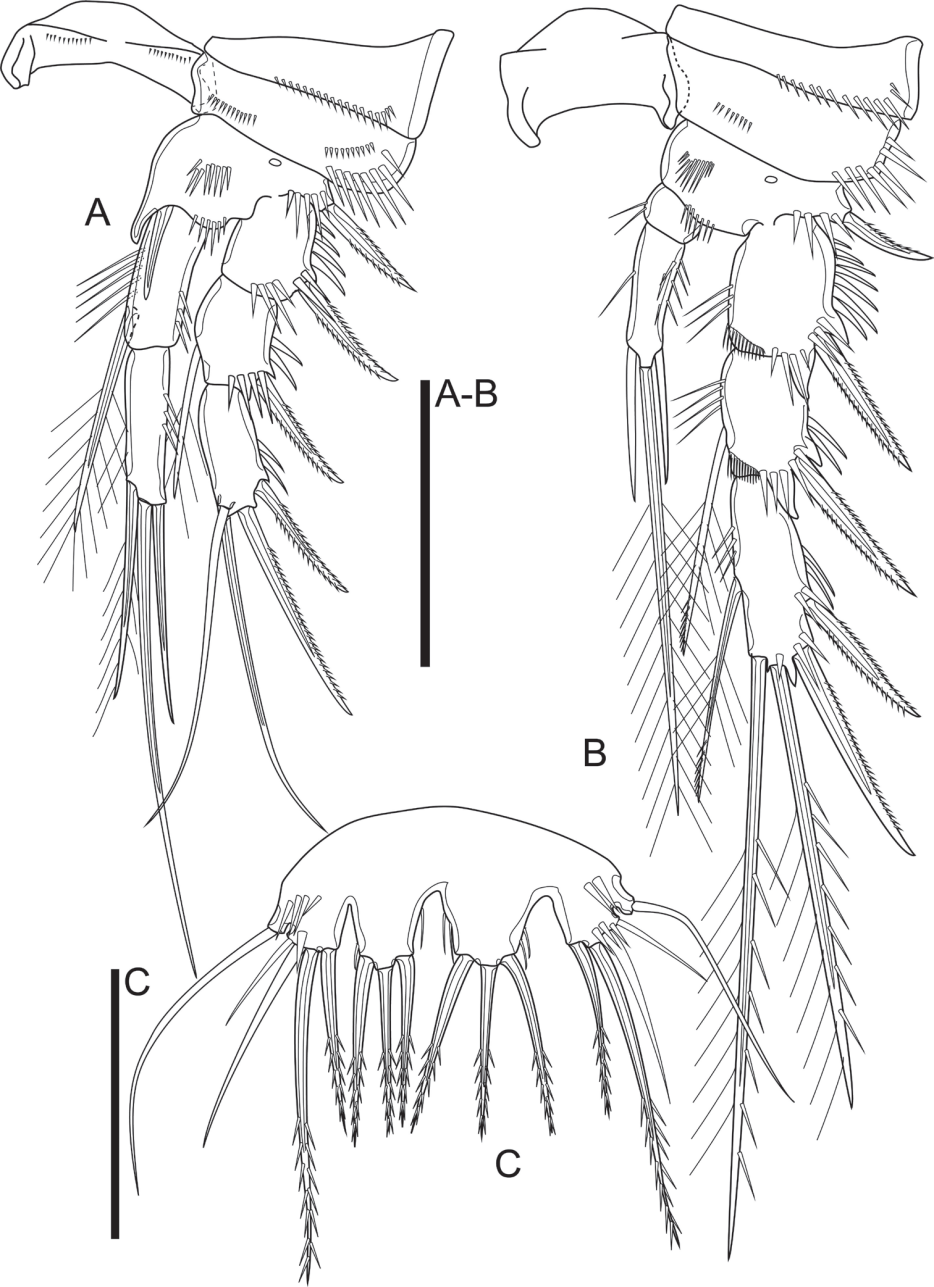
*Cletocamptusthailandensis* sp. nov. male, allotype **A** P1 **B** P2 **C** P5. Scale bars: 50 μm.

P1 (Fig. 10A) as that of female, except for the projection on inner distal corner of basis.

P2 (Fig. 10B) as that of female, except for sexual dimorphic (slightly shorter and stronger) inner seta on Endp-2.

**Figure 11.**
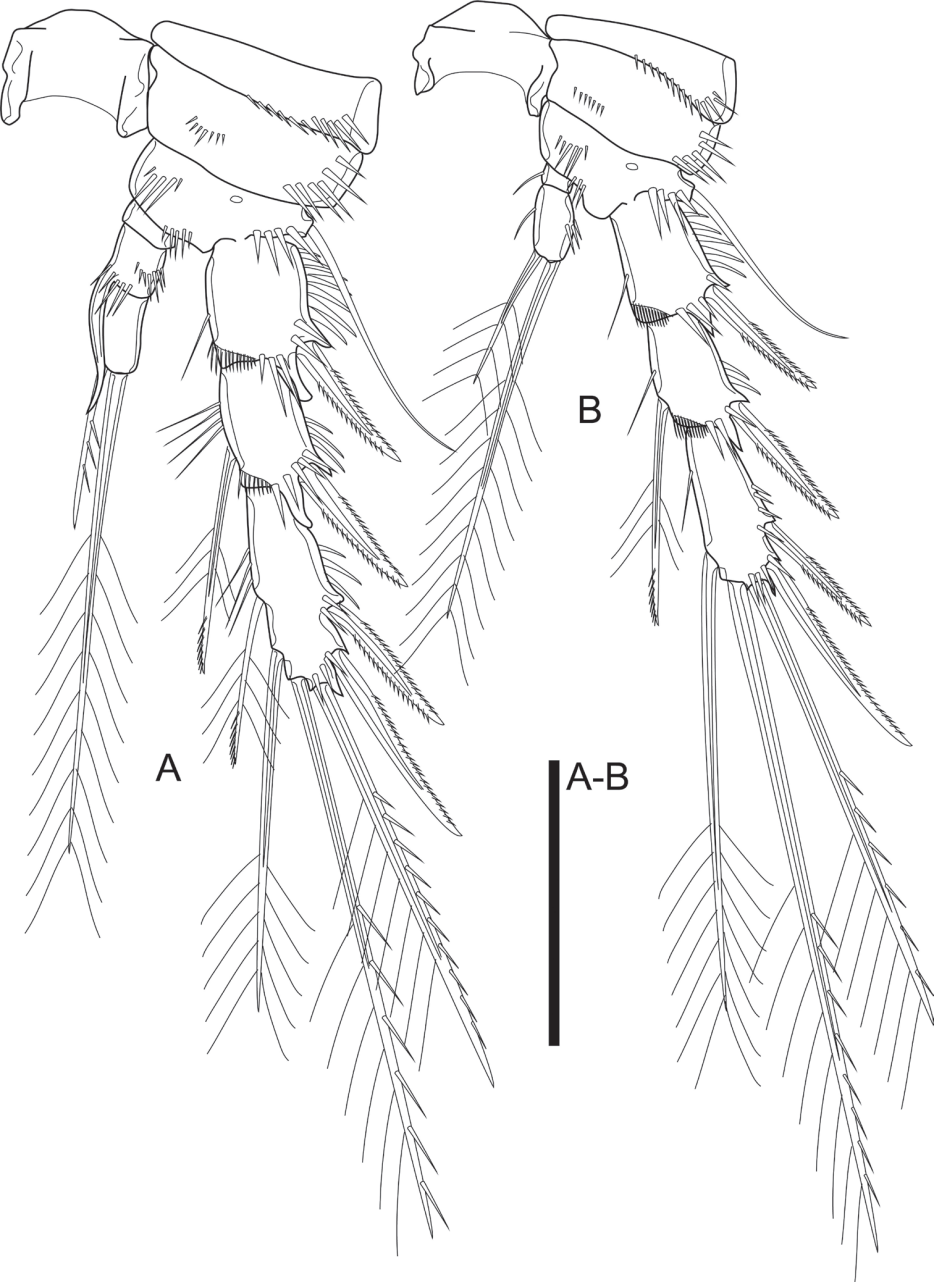
*Cletocamptusthailandensis* sp. nov. male, allotype **A** P3 **B** P4. Scale bar: 50 μm.

P3 (Fig. 11A) as that of female in intercoxal sclerite, praecoxa, coxa, basis, and Exp. Endp three-segmented; Endp-1 short, wider than long; Endp-2 drawn out into apophysis, with row of spinules on outer margin and on frontal surface near base of Endp-3, apophysis surpassing tip of Endp-3; Endp-3 with two apical setae.

P4 (Fig. 11B) as that of female.

Left and right legs of P5 (Fig. 10C) fused medially at base, with medial notch indicating former separation between them; baseoendopod and Exp fused basally, with large notch indicating division between baseoendopodal and exopodal lobes. Baseoendopodal lobe with three pinnate spiniform setae apically, setae subequal in length; exopodal lobe with spinules at base of outermost apical seta, armed with four elements of which three apical ones pinnate and spiniform, outermost smooth and slender. Smooth soft seta on outer margin of basis, accompanied with row of spinules at base.

P6 (Fig. 9A) reduced to simple plate, without armature and ornamentation. Posterior margin smooth.

###### Etymology.

The species name is a noun proposed to reflect the name of the country, ‘Thailand’, where the new species was encountered. It is in the nominative singular, gender masculine.

###### Type locality.

The new species was collected from a water body at the base of an isolated limestone mountain (Fig. 1A, B) that is the distalmost part of the Cha Moon-Cha Mao Mountain Range in the Gong Din Subdistrict, Klaeng District, Rayong Province, Eastern Thailand. The coordinates of the type locality are 12°45'52.96"N, 101°47'55.16"E. The mountain is located beside a monastery known in Thai as ‘Wat Tham Rakang Thong’ (Tham Rakang Thong Monastery). The mountain and the water body are surrounded by a manmade concrete road (Fig. 1C), which separates the mountain and the monastery from the mangrove forests. The water is ca. 20 cm in depth and transparent with a brown color. The bottom of the water body is covered by leaf litter and filamentous algae. On the first sampling occasion, the temperature was 27.4 °C, the pH was 5.48, and the dissolved oxygen level was 5.5 mg L^-1^. The water electroconductivity and total dissolved solids were higher than 3,999 µS cm^-1^ and 2,000 ppm, respectively. On the second occasion, the temperature was 30.0 °C, the pH was 6.53, the electroconductivity was 2,158 µS cm^-1^, and the total dissolved solids were 1,080 ppm. The new species was collected with other zooplankton species, such as *Phyllognathopuspaludosus* (Mrazek, 1893), *Mesocyclopsogunnus* Onabamiro, 1957 (the most dominant), *Apocyclopsborneoensis* Lindberg, 1954, *Tropocyclops* sp., *Nitokra* sp., the rotifer of the genus *Testudinella* Bory de St. Vincent, 1822, the cladoceran of the genus *Leberis* Smirnov, 1989, and ostracods. Aquatic insects belonging to the order Ephemeroptera and the family Chironomidae were also observed.

###### Distribution.

The species has been known from the type locality only.

### ﻿Remarks

The new species was assigned to the genus *Cletocamptus* because of the combination of the following characteristics:

1. a sexually dimorphic rostrum with subdistal ventral spinules,
2. a cephalothorax and prosomite with long spinules along the posterior margin,
3. a sexually dimorphic outer spine on the P2Endp-2,
4. the genital and first abdominal somites being completely fused ventrally, forming a genital double-somite with the dorsal subchitinous rib on the dorsal and lateral surfaces.

The genus has now been placed in the subfamily Cletocamptinae of the family Canthocamptidae, along with *Cletocamptoides* and *Amphibiperita* (Gómez and Yáñez-Rivera 2022). Among these three genera, *Cletocamptus* is the sister group of *Cletocamptoides*, according to phylogenetic research (Gómez and Yáñez-Rivera 2022). The new species and other *Cletocamptus* can be distinguished from *Cletocamptoides* by the following characteristics:

1. the absence of a somatic constriction between the somite,
2. the presence of long spinules on the posterior margin of the cephalothorax and prosomite,
3. the reduced mandibular palp bearing, at most two setae on the free segment and one seta nearby (as opposed to three setae on one well-developed segment),
4. the P4 with two-segmented Endp (reduced to one segment in *Cletocamptoides*).

Recently, 28 species of *Cletocamptus* have been validated. In the valid species, five groups of species can be defined based on the combination of the following female characteristics: the number of setae on the P3Endp-2, the relative length of the caudal ramus, the relative length of the inner apical seta on the P3Endp-2, the shape of the P5, and the number of setae on the P3Exp-3 (Table 2). The first group is composed of *C.albuquerquensis*, *C.dominicanus*, *C.tainoi*, *C.confluens*, *C.chappuisi*, and *C.trichotus*, which bear relatively long caudal rami (> 2× as long as wide), P3Endp-2 with five setae, P3Endp-2 with inner apical seta reaching the middle of the outer one, P5 exopodal lobe highly fused to the baseoendopodal lobe in the P5 of both males and females (baseoendopodal and exopodal lobes separated by small notch), and P3Exp-3 with five setae. In many species, the P1Endp-1 is relatively short, reaching the middle of Exp-2 at most, and the caudal seta IV and V are fused at the base. The second group comprises *C.pilosus* and *C.retrogressus*, which bear relatively long caudal rami (> 2× as long as wide), P3Endp-2 with five setae, P3Exp-3 with six setae, and P5 with a large notch between the exopodal and baseoendopodal lobes in both males and females. The inner apical seta of P3Endp-2 reaches the middle of the outer one in *C.retrogressus*, but it is shorter in *C.pilosus*. Furthermore, caudal seta IV and V are fused at the base in the former but separated in the latter. The third group is composed of *C.gomezi*, *C.mongolicus*, *C.feei*, *C.gravihiatus*, and *C.affinis*, which, in general, bear relatively long caudal rami (> 2× as long as wide), P3Endp-2 with short inner apical seta not reaching the middle of the outer one, P5 with a large notch between the exopodal and baseoendopodal lobes in both males and females, and P3Exp-3 with five setae. In this group, a reduction of either the number or the length of the inner seta of the P3Endp-2 was observed, resulting in variations in the number of setae on the P3Endp-2. In *C.gomezi*, *C.mongolicus*, *C.gravihiatus* and *C.affinis*, there are five setae, whereas there are four in *C.feei*. Even if the number of inner setae is not reduced, the inner and inner apical seta are relatively shorter than those of most species of the first and the second groups. The fourth group is composed of *C.stimpsoni*, *C.goenchim*, and *C.koreanus*, which bear relatively short caudal rami (generally, ca. 1.5–1.7× as long as wide), P3Endp-2 with five setae, P3Endp-2 with an inner apical seta reaching the middle of the outer one (except in *C.stimpsoni*), P5 with a large notch between the exopodal and baseoendopodal lobes in both males and females, and P3Exp-3 with six setae. It has been found that P1Endp-1 reaches the distal margin of the Exp-2. The last group comprises *C.assimilis*, *C.axi*, *C.cecsurirensis*, *C.deborahdexterae*, *C.fourchensis*, *C.levis*, *C.nudus*, *C.samariensis*, *C.schmidti*, *C.sinaloensis*, *C.spinulosus*, and *C.tertius*, which, in general, bear relatively short caudal rami (mostly ca. 1.5× as long as wide), P3Endp-2 with three setae, P3Endp-2 with an inner apical seta not reaching the middle of the outer one (except *C.spinulosus*), P5 with a large notch between the exopodal and baseoendopodal lobes in both males and females, and P3Exp-3 with six setae in *C.nudus*, *C.samariensis*, and *C.schmidti* but five in the rest. In this group, the P1Endp-1 is relatively short, reaching the middle of Exp-2 at most, and the lateral seta of the praecoxal arthrite of the maxillule is robust and spinulose in many species, including *C.assimilis*, *C.axi*, *C.cecsurirensis*, *C.deborahdexterae*, *C.fourchensis*, *C.levis*, *C.nudus*, *C.samariensis*, *C.schmidti*, *C.sinaloensis*, and *C.tertius*. Only *C.spinulosus* has slender seta.

**Table 2.** Comparison of characters among five groups of species of the genus *Cletocamptus*. The superscripts indicate the feature that is less common in the group, and the species possessing it is listed in the note below the table.

Characters	Species group 1	Species group 2	Species group 3	Species group 4	Species group 5
**Female**					
1. Relative length of the caudal ramus	ca. 3–4 or 2^A^	ca. 2^A^ or 3^B^	ca. 3 or 2^A^	ca. 1.5–1.7	ca. 1.5–1.7 or ca. 1.1^C^ or 2^A^ or 3^B^
2. Caudal seta IV and V	Fused at base or separated^D^	Fused at base^E^ or separated^D^	Separated	Separated	Separated
3. Armature formula of number of setae/spines on P2–P4Endp-2	3.5.2 or 3.3.2^F^ or 4.5.2^G^	4.5.2	3.5.2 or 4.5.2^H^ or 3.4.2^I^	3.5.2	3.3.2
4. Armature formula of number of setae/spines on P2–P4Exp-3	5.5.4	5.6.5	5.5.4 or 5.5.5^J^	5.6.5	5.5.4 or 5.6.5^K^
5. Length ratio of inner apical seta and outer apical seta of P3Endp-2	ca. 0.6–0.8	ca. 0.3–0.8	ca. 0.2	ca. 0.5–0.6	ca. 0.3–0.5
6. Notch between exopodal and baseoendopodal lobes	Small and shallow	Large	Large	Large	Large
**Male**					
7. Number of setae/spines on P2Endp-2	3	4	3 or 4^L^	3	3
8. Endp-2 and Endp-3 of P3	Fused	Separated	Separated	Separated	Separated
9. Modification of P3Exp-1 in comparing to that of female	Elongated or not elongated^M^	Elongated or not elongated^M^	Slightly elongated	Not elongated	Elongated or not elongated^M^

**Note**: ^A^ = in *C.dominicanus*, *C.trichotus*, *C.pilosus*, *C.gomezi*, *C.axi*, and *C.schmidti*. ^B^ = in *C.retrogressus*, *C.tertius* and *C.spinulosus*. ^C^ = in *C.cecsurirensis*. ^D^ = in *C.trichotus* and *C.pilosus*. ^E^ = in *C.retrogressus*. ^F^= in *C.confluens*^G^ = in *C.trichotus*. ^H^ and ^L^ = in *C.gomezi*. ^I^ and ^J =^ in *C.feei*. ^K^ = in *C.nudus*, *C.samariensis*, and *C.schmidti*. ^M^ = in *C.dominicanus*, *C.retrogressus*, *C.nudus*, *C.samariensis*, and *C.schmidti*.

Based on the above criteria, the new species belongs to the fourth group, and its most closely related species are *C.koreanus* and *C.goenchim*, corresponding to their geographical distribution. According to the characteristic of the relative length of the inner apical seta on P3Endp-2, *C.pilosus* and *C.stimpsoni* could be included in the third group. However, the setae on P3Exp-3 are more numerous in *C.pilosus* and *C.stimpsoni* (with six setae) than in the third group (with five setae). Furthermore, as the reduction of the inner seta and inner apical seta on P3Endp-2 is commonly observed in the members of the third group, the reduction in length of the inner apical seta is probably convergent among *C.pilosus*, *C.stimpsoni*, and the members of the third group.

Previously, *C.koreanus* and *C.goenchim* were described in Korea and India, respectively (Chang 2013; Gómez et al. 2013). The new species and the two above-mentioned ones share several characteristics, including the length ratio of Endp-1 and Endp-2 of P1, the relative length of P1Endp and P1Exp, the integumental ornamentation of the cephalothorax, the armament and ornamentations of P1–P4 in both sexes and the female P5, the ornamentation of the anal operculum, the relative length of the caudal ramus, the number of abexopodal setae on the antennary allobasis, the number of setae on the antennary exopod and mandibular palp, and the shape of the lateral seta on the praecoxal arthrite of the maxillule. However, based on the author’s knowledge, there is no characteristic unique to these three Asian *Cletocamptus*.

A finely detailed examination showed that the new species can be distinguished from *C.koreanus* and *C.goenchim*. The exopodal lobe of the male P5 of the new species has four elements, whereas it has three elements in *C.koreanus* and *C.goenchim*. The lateral surface of all abdominal segments is ornamented with a row of moderate long spinules in the new species, which are absent in *C.koreanus* and *C.goenchim*. Between the caudal dorsal seta VII and the row of spinules near the caudal seta VI of the females, there is an additional row of spinules on the inner margin that is absent in *C.koreanus* and *C.goenchim*. The aesthetasc on the fourth segment of the female antennule is relatively longer in the new species, with ca. 45% of the length of the aesthetasc surpassing the tip of the antennule, whereas it is relatively shorter in *C.koreanus* and *C.goenchim*, where less than 40% of the length of the aesthetasc surpasses the tip of the antennule. The new species lacks the spinular row at the base of the basal outer seta of the female P5 that is present in *C.koreanus* and *C.goenchim*. There are few spinules at the base of the basal outer seta of the male P5 in the new species and *C.koreanus*, but they are absent in *C.goenchim*. Furthermore, there are spinule rows on the frontal surface of the male P3Endp-2 that are absent in *C.koreanus* and *C.goenchim*.

### ﻿Key to the female of *Cletocamptus*

The description of *C.chappuisi* was done, based only on the male by Chappuis (1933) and Gómez et al. (2017). So, it was not included in this key. However, it would be suggested that *C.chappuisi* belongs to the species group 1, according to the chaetotaxies of P2–P4Endp-2 and P2–P4Exp-3, along with the shape and chaetotaxy of caudal rami.

**Table:** 

1	P5 exopodal and baseoendopodal lobes highly fused, barely separated by small shallow notch or without notch; caudal rami ca. 3–4× as long as wide (ca. 2× as long as wide in some species); caudal seta IV and V fused at base or separated; P2–P4Endp-2 with 3 or 4, 5, 2 elements (= setae and spines), respectively; P2–P4Exp-3 with 5, 5, 4 elements, respectively; P3Endp-2 with inner apical seta reaching or surpassing middle of outer apical seta, species group 1	**2**
–	P5 with large notch between exopodal and baseoendopodal lobes; caudal rami relatively short, generally ca. 1.1–2× as long as wide (> 3× as long as wide in some species); caudal seta IV and V fused at base or separated	**6**
2	A2Exp with 3 setae	**3**
–	A2Exp with 1 setae	**4**
3	Caudal seta IV and V fused at base; P2Endp-2 with 3 elements	***C.albuquerquensis***
–	Caudal seta IV and V separated; P2Endp-2 with 4 elements	***C.trichotus***
4	Mandible with 2 setae on palp and 1 seta arising nearby	***C.tainoi***
–	Mandible with 3 setae on palp	**5**
5	Caudal rami ca. 2× as long as wide; exopodal and baseoendopodal lobes with 5 and 6 marginal setae, respectively	***C.dominicanus***
–	Caudal rami ca. 3× as long as wide; exopodal and baseoendopodal lobes with 4 and 6 marginal setae, respectively	***C.confluens***
6	P2–P4Endp-2 with 4, 5, 2 elements, respectively; P2–P4Exp-3 with 5, 6, 5 elements, respectively; P1 with Endp surpassing tip of Exp, species group 2	**7**
–	P2–P4Endp-2 with 3 or 4, 4 or 5, 2 elements, respectively; P2–P4Exp-3 with 5, 5, 4 or 5 elements, respectively; relative length of caudal rami ca. 2–3× as long as wide; inner apical seta on P3Endp-2 relatively short, doing not surpass proximal sixth of outer apical seta, species group 3	**8**
–	P2–P4Endp-2 with 3, 5, 2 elements, respectively; P2–P4Exp-3 with 5, 6, 5 elements, respectively; relative length of caudal rami ca. 1.5–1.7× as long as wide; P3Endp-2 with inner apical seta surpassing proximal third of outer apical seta and generally reaching middle of outer apical seta; P1 with Endp-1 subequal in length to Endp-2, species group 4	**12**
–	P2–P4Endp-2 with 3, 3, 2 elements, respectively; P2–P4Exp-3 with 5, 5, 4 or 5, 6, 5 elements, respectively; inner apical seta on P3Endp-2 reaching proximal third of outer apical seta at most (reaching middle of outer apical seta in some species); P1 with Endp-1 shorter than Endp-2, species group 5	**15**
7	Caudal seta IV and V fused at base; caudal rami 2.5× as long as wide; P1 with Endp-1 longer than Endp-2	***C.retrogressus***
–	Caudal seta IV and V separated; caudal rami at most 2× as long as wide; P1 with Endp-1 shorter than Endp-2	***C.pilosus***
8	P1Endp-1 with inner seta	**9**
–	P1Endp-1 without inner seta	**10**
9	P2–P4Endp-2 with 4, 5, 2 elements, respectively	***C.gomezi***
–	P2–P4Endp-2 with 3, 5, 2 elements, respectively	***C.affinis***
10	P2–P4Endp-2 with 3, 4, 2 elements, respectively	***C.feei***
–	P2–P4Endp-2 with 3, 5, 2 elements, respectively	**11**
11	Antennule six-segmented	***C.mongolicus***
–	Antennule seven-segmented	***C.gravihiatus***
12	Inner apical seta on P3Endp-2 reduced, doing not reach the proximal fourth of outer apical seta; posterior margin of cephalic shield and prosomite 2–3 with short spinules; mandible with 2 setae on palp only	***C.stimpsoni***
–	Inner apical seta on P3Endp-2 normally develops, reaching middle of outer apical seta; posterior margin of cephalic shield and prosomite 2–3 with long spinules; mandible with 2 setae on palp and 1 seta arising near palp	**13**
13	Caudal rami with 4 transverse rows of spinules on inner margin; lateral surfaces of third and fourth urosomites (second and third abdominal somites) with transverse rows of moderately long spinules (these spinules as long as those of medial spinule row on ventral surface); female P5 without spinule row at base of basal seta	***C.thailandensis* sp. nov.**
–	Caudal rami with 3 transverse rows of spinules on inner margin; lateral surfaces of third and fourth urosomites without transverse rows of moderately long spinules; female P5 with spinule row at the base of basal seta	**14**
14	Maxilliped with basis ca. 3× as long as wide, posteriormost spinule row on ventral surface of fourth urosomite continuous	***C.koreanus***
–	Maxilliped with basis ca. 2.5× as long as wide; posteriormost spinule row on ventral surface of fourth urosomite medially interrupted	***C.goenchim***
15	P2–P4Exp-3 with 5, 6, 5 elements, respectively	**16**
–	P2–P4Exp-3 with 5, 5, 4 elements, respectively	**18**
16	Anal operculum without spinule on distal margin	***C.nodus***
–	Anal operculum with spinule on distal margin	**17**
17	Mandible with 2 setae on palp only	***C.samariensis***
–	Mandible with 2 setae on palp and 1 short seta arising nearby	***C.schmidti***
18	Anal operculum without spinule on distal margin	***C.fourchensis***
–	Anal operculum with spinules on distal margin	**19**
19	Caudal rami ca. 3× as long as wide	**20**
–	Caudal rami ca. 1.1–2× as long as wide	**21**
20	Posterior margin of cephalic shield with long spinule dorsally and laterally; lateral seta on praecoxal arthrite strong; P6 with 3 setae	***C.tertius***
–	Posterior margin of cephalic shield with small spinules laterally, bare or with few spinules dorsally; lateral seta on praecoxal arthrite slender; P6 with 2 setae	***C.spinulosus***
21	Caudal rami ca. 1.1× as long as wide	***C.cecsurirensis***
–	Caudal rami ca. 1.5–1.6× as long as wide	**22**
–	Caudal rami ca. 1.7–2× as long as wide	**24**
22	A2Exp with 2 setae; P6 with 1 seta; anal operculum with two rows of strong spinules	***C.sinaloensis***
–	A2Exp with 3 setae; P6 with 1 or 2 setae	**23**
23	P6 with 1 seta; Cephalic shield with long spinules along lateral margin, with or without smaller spinules along posterior-dorsal and dorso-lateral margin (smaller spinules shorter than those of second and third prosomites	***C.deborahdexterae***
–	P6 with 2 setae; Cephalic shield with long spinules along posterior margin dorsally and laterally	***C.levis***
24	Caudal rami with transverse row of strong spinules on medial margin	***C.axi***
–	Caudal rami without transverse row of strong spinules on medial margin	***C.assimilis***

### ﻿Key to the male of *Cletocamptus*

**Table:** 

1	P5 exopodal and baseoendopodal lobes, barely separated by small shallow notch or without notch; caudal rami ca. 3–4× as long as wide (ca. 2× as long as wide in some species); caudal seta IV and V fused at base or separated; P2–P4Endp-2 with 3 or 4, 5, 2 elements, respectively; P2–P4Exp-3 with 5, 5, 4 elements, respectively; P3Endp-2 and Endp-3 fused, species group 1	**2**
–	P5 with large notch between exopodal and baseoendopodal lobes; caudal rami relatively short, generally ca. 1.1–2× as long as wide (≥ 3× as long as wide in some species); caudal seta IV and V fused at base or separated; P3Endp-2 and Endp-3 separated	**7**
2	A2Exp with 3 setae	**3**
–	A2Exp with 1 setae	**4**
3	Caudal seta IV and V fused at base; P3Endp-3 with inner apical seta ca. 2× as long as outer apical one	***C.albuquerquensis***
–	Caudal seta IV and V separated; two apical setae on P3Endp-3 subequal in length	***C.trichotus***
4	Anal operculum without spinule on distal margin	***C.chappuisi***
–	Anal operculum with spinule on distal margin	**5**
5	Mandible with 3 setae on palp	**6**
–	Mandible with 2 setae on palp and 1 seta arising nearby	***C.tainoi***
6	Caudal rami ca. 4× as long as wide; P3Endp-2 modified, with 2 strong dentiform projections; P5 exopodal lobe with 3 marginal setae	***C.confluens***
–	Caudal rami ca. 2 times as long as wide; P3 with Endp-2 fused to Endp-3, with apophysis; P5 exopodal lobe with 4 marginal setae	***C.dominicanus***
7	Caudal rami ca. 2–3× as long as wide; P2Endp-2 with 4 elements; P2–P4Exp-3 with 5, 6, 5 elements, respectively, P1Endp surpassing Exp; P1 with Endp-1 unequal in length to Endp-2, species group 2	**8**
–	Caudal rami ca. 3× as long as wide (ca. 2× as long as wide in some species); P2Endp-2 with 3 or 4 elements; P2–P4Exp-3 with 5, 5, 4 elements, respectively; P1 with Endp-1 longer or subequal in length to Endp-2, species group 3	**9**
–	Caudal rami ca. 1.5–1.7× as long as wide; P2Endp-2 with 3 or 4 elements; P2–P4Exp-3 with 5, 6, 5 elements, respectively; P1 with Endp-1 subequal in length to Endp-2, species group 4	**13**
–	Caudal rami ca. 1.1–1.7× as long as wide (2 or 3× as long as wide in some species); P2Endp-2 with 3 elements; P2–P4Exp-3 with 5, 6, 5 or 5, 5, 4 elements, respectively; P1 with Endp-1 shorter than Endp-2, species group 5	**16**
8	Caudal seta IV and V fused at base; caudal rami at least 2.5× as long as wide; P1 with Endp-2 longer than Endp-1	***C.retrogressus***
–	Caudal seta IV and V separated; caudal rami ca. 2× as long as wide; P1 with Endp-2 shorter than Endp-1	***C.pilosus***
9	Caudal rami with 7 setae	***C.mongolicus***
–	Caudal rami with 6 setae	**10**
10	P2Endp-2 with 4 elements	***C.gomezi***
–	P2Endp-2 with 3 elements	**11**
11	P1Endp-1 with inner seta	***C.affinis***
–	P1Endp-1 without inner seta	**12**
12	P3Endp-3 with inner seta surpassing tip of apophysis	***C.feei***
–	P3Endp-3 with inner seta doing not surpass tip of apophysis	***C.gravihiatus***
13	P2Endp-2 with 4 elements; mandible with 2 setae on palp only	***C.stimpsoni***
–	P3Endp-2 with 3 elements; mandible with 2 setae on palp and 1 seta arising nearby	**14**
14	P5 exopodal lobe with 4 marginal setae	***C.thailandensis* sp. nov.**
–	P5 exopodal lobe with 3 marginal setae	**15**
15	Baseoendopodal lobe with 4 setae, or often 3 or 4 setae asymmetrically	**C.koreanus**
–	Baseoendopodal lobe with 3 setae consistently	***C.goenchim***
16	P2–P4Exp-3 with 5, 6, 5 elements, respectively; P3Exp curved; P3Exp-1 elongated, comparing to that of female	**17**
–	P2–P4Exp-3 with 5, 5, 4 elements, respectively; P3Exp straight; P3Exp-1 similar to that of female	**19**
17	Anal operculum without spinule on distal margin; Left and right P5 distinct	***C.nodus***
–	Anal operculum with spinule on distal margin	**18**
18	Mandible with 2 setae on palp and lacking seta arising nearby	***C.samariensis***
–	Mandible with 2 setae on palp and 1 short seta arising nearby	***C.schmidti***
19	Caudal rami at least 3× as long as wide	**20**
–	Caudal rami ca. 1.1–2× as long as wide	**21**
20	Medial element on P1 basis spiniform; lateral seta on praecoxal arthrite strong; outer spine on P2Endp-2 strongly curved and robust; lateral seta on A2Exp reduced, shorter than outer apical seta	***C.tertius***
–	Medial element on P1 basis setiform; lateral seta on praecoxal arthrite slender; outer spine on P2Endp-2 slightly curved and thin; lateral seta on A2Exp normal developed, as long as outer apical seta	***C.spinulosus***
21	Left and right P5 distinct	***C.cecsurirensis***
–	Left and right P5 fused	**22**
22	Caudal rami ca.1.5–1.7× as long as wide	**23**
–	Caudal rami ca. 2× as long as wide	***C.axi***
23	A2Exp with 2 setae; fifth urosomite without medial row of moderately long spinules	***C.sinaloensis***
–	A2Exp with 3 setae; fifth urosomite with medial row of moderately long spinules	**24**
24	Posterior margin of cephalic shield with few small spinules dorsally, with long spinules laterally	***C.levis***
–	Posterior margin of cephalic shield with long spinules dorsally and laterally	**25**
25	P5 exopodal lobe with longest seta ca. 7× as long as the outermost seta; outer spine on P2Endp-2 strongly curved and robust	***C.fourchensis***
–	P5 exopodal lobe with longest seta ca. 2.9× as long as the outermost seta	***C.deborahdexterae***

## ﻿Discussion

Among the representatives of the genus *Cletocamptus*, *C.deitersi* is the most problematic in that it expresses a high degree of morphological variation (Gómez et al. 2004). The species has so far been recorded in North and Central America, as well as in India, China, Ethiopia, Hawaii, Australia, Iran, and Malaysia (see the list of references in Gómez et al. 2004). However, recent molecular and morphological studies have proved that *C.deitersi* is a mixture of different species (Rocha-Olivares et al. 2001; Gómez et al. 2004, 2007; Gómez and Gee 2009), and its cosmopolitan distribution is the result of the insufficient description and inadequate illustration of Richard’s original description of the species separation of *C.deitersi*. Recently, it has been recognized as *species inquirendae* (Gómez et al. 2004). In Asia, Ranga Reddy and Radhakrishna (1979) reported *C.deitersi* in India without a description or any comments on the morphology of the specimens, while Tai and Song (1979) reported it in China, with a short description of the female. Gómez et al. (2013) pointed out that Ranga Reddy and Radhakrishna (1979) identified the Indian specimens on the basis of the revision of Lang (1948) and the description of Hamond (1973), who reported the presence of *C.deitersi* in Australia. Gómez et al. (2013) also argued that the female P3 of the Australian *C.deitersi* is likely similar to that of *C.brehmi*, which has been considered by Chappuis (1933) and Kiefer (1936) as a synonym of *C.deitersi*. Based on the viewpoint of Gómez et al. (2013), the new species shows a close affinity to the Indian *C.deitersi* in having five setae on its P3Endp-2. However, we found that Kiefer’s (1936) and Hamond’s (1973)*C.deitersi* share the presence of four setae on the exopodal lobe of the female P5, which is different from that of the Thai *Cletocamptus*. Based on the illustrations of Daday (1902) and Chappuis (1934) adopted by Lang (1948), the new species differs from Daday’s (1902)*C.deitersi*, as the inner apical seta of the female P3Endp-2 does not reach the middle of the outer apical one in the latter but reaches the middle of the inner one in the new species. We believe that Daday’s (1902)*C.deitersi* is morphologically related to the member of the third group of valid species because it has a short inner apical seta on the P3Endp-2 and a relatively long caudal ramus. Furthermore, whereas the outer spine of the male P2Endp-2 is straight and slim in the new species, it is curved and shortened in Chappuis’s (1934)*C.deitersi*. The curved and shortened spine of the male P2Endp-2 of Chappuis’s (1934)*C.deitersi* seems similar to that of *C.levis*, *C.fourchensis*, *C.sinaloensis*, and *C.goenchim*.

The type locality is the water body at the base of an isolated limestone mountain. Within it, two microhabitats could be defined: a submerged filamentous algal mat and an area of leaf litter. A greater number of specimens was collected from the filamentous algal mat, indicating the habitat preference of the aerated zone of the new species. It seems like that of *C.gomezi*, which is absent in the sandy bottom but present in the filamentous algal mat of *Ruppiamaritima* L. and *Chara* sp. (Suárez-Morales et al. 2013).

The global distribution and the high degree of polymorphism of *C.deitersi* have previously been mentioned. Gómez et al. (2004), however, suggested that it is because of the high degree of intraspecific variability of *Cletocamptus* species and the morphologically similar among the specimens attributed to *C.deitersi*. After the molecular study of the American and Mexican specimens confirmed the hypothesis of several authors, showing clearly that different species had been identified as *C.deitersi* (Rocha-Olivares et al. 2001), it has recently been accepted that *C.deitersi* is a species complex, consists of morphologically indistinguishable sibling species, that cannot be differentiated, based on Richard’s original description (Gómez et al. 2004). Because the description of such records was done on the conservative features with which they are insufficient for species separation (see list of contributions in Gómez et al. 2004), several records of *C.deitersi* around the globe have now been considered *species inquirenda* (Gómez et al. 2004). Many new species have later been elevated for the specimens previously identified as *C.deitersi* (e.g., Gómez et al. 2004; Gómez 2005; Gómez and Gee 2009). However, as morphological differences can occur between the partial populations of a cosmopolitan species (Mielke 2000a, b), and information about the variability of the genuine *C.deitersi* remains unknown, it raises doubt about whether they are independent species or subspecies. So, the knowledge of the variability and the full description of the genuine *C.deitersi* from the type locality is needed for further taxonomic work on the status clarification of *C.deitersi*, as well as the recently described species.

## Supplementary Material

XML Treatment for Cletocamptus thailandensis
